# Supplementary material for: Bacteriophage DNA induces an interrupted immune response during phage therapy in a chicken model
Source: Nat Commun. 2024 Mar 13;15:2274. doi: 10.1038/s41467-024-46555-7 (PMC10937645; doi:10.1038/s41467-024-46555-7)
Supplement: Supplementary file 1 — Supplementary Information [file 41467_2024_46555_MOESM1_ESM.pdf]

Supplementary Table S1. Blood parameters of mice used for isolation of splenocytes.

ID	Animal	WBC (10^9/L)	LYM%	MON%	NEU%	EOS%	BASO%	LYM# (10^9/L)	MON# (10^9/L)	NEU# (10^9/L)
1	Mice	9.48	82.66	6.89	8.95	0.9	0.6	6.918	0.576	0.751
2	Mice	9.42	80.23	9.53	8.79	0.93	0.52	6.506	0.772	0.715
3	Mice	8.67	79.04	9.51	8.31	3.02	0.12	6.046	0.727	0.637
4	Mice	8.53	79.01	9.03	9.48	2.46	0.02	6.131	0.7	0.738
5	Mice	9.74	73.54	11.8	10.36	3.08	1.22	6.736	1.08	0.951
6	Mice	8.03	73.66	12.97	7.93	5.02	0.42	5.17	0.91	0.559
7	Mice	9.44	71.83	11.11	13.38	3.39	0.29	3.993	0.617	0.746
8	Mice	8.92	62.75	21.7	12.93	2.24	0.38	5.747	1.987	1.187
9	Mice	8.70	68.92	16.82	11.68	1.76	0.82	5.458	1.332	0.927
10	Mice	8.60	67.48	19.05	11.36	1.69	0.42	5.222	1.474	0.882
**ID**	**Animal**	**EOS# (10^9/L)**	**BASO# (10^9/L)**	**RBC (10^{12}/L)**	**HGB (g/dL)**	**HCT (%)**	**MCV (fL)**	**MCH (pg)**	**MCHC (g/dL)**	**CRP (µg/mL)**
1	Mice	0.075	0.05	9.46	15.3	35	47	17.2	35.5	1.17
2	Mice	0.075	0.042	9.66	15.4	36.2	47.5	16.9	35.3	2.1
3	Mice	0.231	0.009	9.31	15.9	34.5	47.1	17	36	2.2
4	Mice	0.19	0.001	9.23	15.8	34.6	47	17.1	35.6	2.11
5	Mice	0.282	0.111	6.71	12.2	35.8	48.5	18.1	30.2	1.8
6	Mice	0.352	0.029	8.92	15.2	33.3	47.4	17	35.6	1.7
7	Mice	0.188	0.016	7.24	12.9	36.7	41	17.8	31.3	2.15
8	Mice	0.205	0.034	6.76	11.8	40.8	43.9	17.5	30.1	2.21
9	Mice	0.139	0.064	6.83	11.7	35	46.7	17.1	35.8	1.9
10	Mice	0.13	0.032	6.54	11.7	33.8	46.5	17.8	31.1	2.01

Reference values	
**WBC**	0.80-10.60 10^9/L
**LYM %**	40.00 – 92.00 %
**MON %**	1.50 – 18.00 %
**NEU %**	7.30 – 50.00 %
**EOS %**	0.00 – 7.00 %
**BASO %**	0.00 – 1.50 %
**LYM**	0.600 – 8.200 10^9/L
**MON**	0.050 – 1.400 10^9/L
**NEU**	0.400 – 3.600 10^9/L
**EOS**	0.00 – 0.510 10^9/L
**BASO**	0.00 – 0.120 10^9/L

Reference values	
**RBC**	6.50 – 11.50 10^{12}/L
**HGB**	11.00 – 15.5 g/dL
**HCT**	35.00 – 50.00 %
**MCV**	41.00 – 52.00 fL
**MCH**	13.0 – 18.0 pg
**MCHC**	30.0 – 35.0 g/dL

**CRP**	1.12 – 2.68 µg/ml

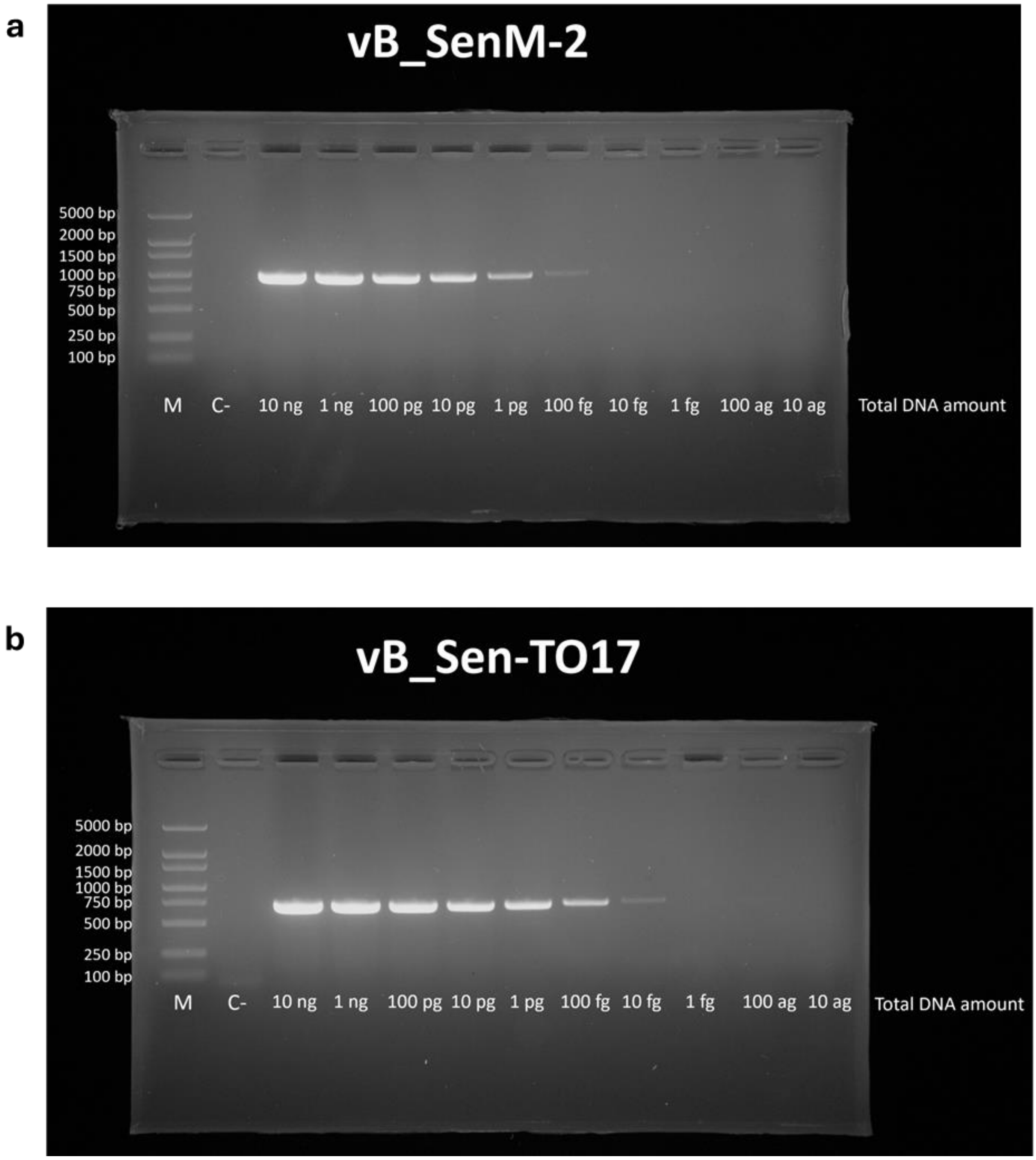

Supplementary Figure S1. Determination of sensitivity of the PCR assay used for detection of DNAs from bacteriophages. Primers specific for DNA regions of bacteriophages vB_SenM-2 (a) and vB_Sen-TO17 (b) were used. Various amounts of template DNA (indicated below corresponding wells) were used in PCR, and reaction products were separated during agarose gel electrophoresis. Lane M contains molecular weight marker (with DNA sizes shown on the left), and lane C- indicates a control without template DNA. The experiment was repeated 3 times with the same results obtained each time.

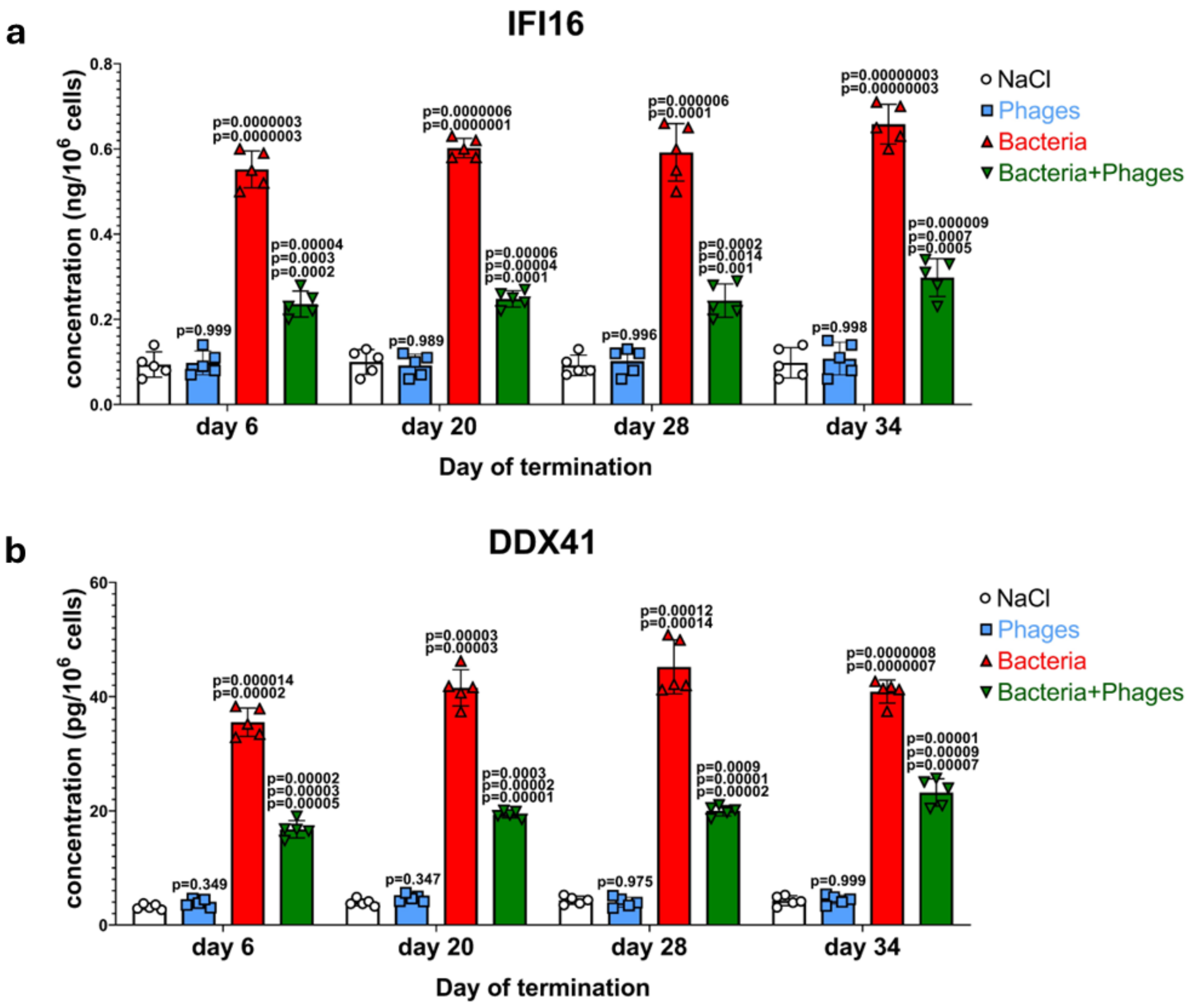

Supplementary Figure S2. Infection of chickens with *S. enterica*, but not administration of the phage cocktail, cause full activation of the elevations of levels of IFI16 and DDX41. Levels of IFI16 (a) and DDX41 (b) were determined by ELISA in samples of blood obtained as indicated in Figure 1 (in the main article). Results correspond to samples obtained from chickens (n=5 for each subgroup) treated with NaCl (white columns with circles representing individual results), phages (blue columns with squares), bacteria (red columns with triangles), and bacteria and phages (green columns with inverted triangles), and are mean values with error bars representing SD. For statistical analyses, the normality of the distribution of variables was checked with the Kolmogorov–Smirnov test and the homogeneity of the variances with Levene's test. Once both assumptions were met, the analysis was carried out using ANOVA and *post-hoc* Tukey's test. Otherwise (one or both assumption(s) was/were not met), the Kruskal–Wallis test and *post-hoc* Dunn test were applied (see the Source Data file for details). The p values obtained in the statistical analyses are indicated above the columns; the green columns (chickens treated with bacteria and phages) are marked with three numbers, indicating p values vs. the groups representing chickens treated with NaCl (lower value), phages (middle values), and bacteria (upper values); consequently, red and blue columns are marked with two and one p value(s), respectively, indicating comparisons with the results shown in columns located to the left of them on the panel. Statistically significant differences were considered when $p<0.05$. Detailed results demonstrated in this figure are included in the Source Data file.

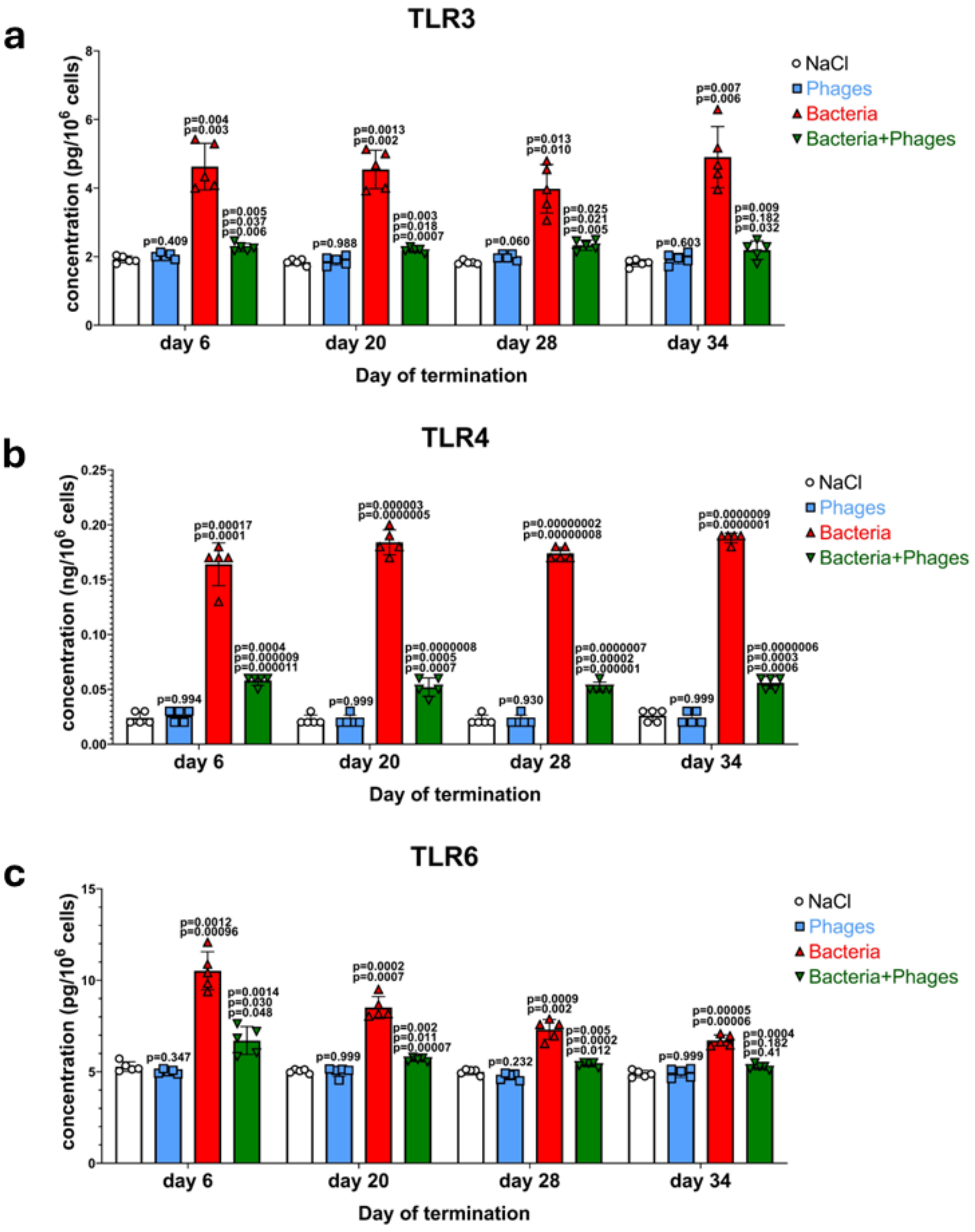

Supplementary Figure S3. Infection of chickens with *S. enterica*, but not administration of the phage cocktail, cause elevations of levels of TLR3, TLR4, and TLR6. Levels of TLR3 (a), TLR4 (b), and TLR-6 (c) were determined by ELISA in samples of blood obtained as indicated in Figure 1 (in the main article). Results correspond to samples obtained from chickens (n=5 for each subgroup) treated with NaCl (white columns with circles representing individual results), phages (blue columns with squares), bacteria (red columns with triangles), and bacteria and phages (green columns with inverted triangles), and are mean values with error bars representing SD. For statistical analyses, the normality of the distribution of variables was checked with the Kolmogorov–Smirnov test and the homogeneity of the variances with Levene's test. Once both assumptions were met, the analysis was carried out using ANOVA and *post-hoc* Tukey's test. Otherwise (one or both assumption(s) was/were not met), the Kruskal–Wallis test and *post-hoc* Dunn test were applied (see the Source Data file for details). The p values obtained in the statistical analyses are indicated above the columns; the green columns (chickens treated with bacteria and phages) are marked with three numbers, indicating p values vs. the groups representing chickens treated with NaCl (lower value), phages (middle values), and bacteria (upper values); consequently, red and blue columns are marked with two and one p value(s), respectively, indicating comparisons with the results shown in columns located to the left of them on the panel. Statistically significant differences were considered when $p<0.05$. Detailed results demonstrated in this figure are included in the Source Data file.

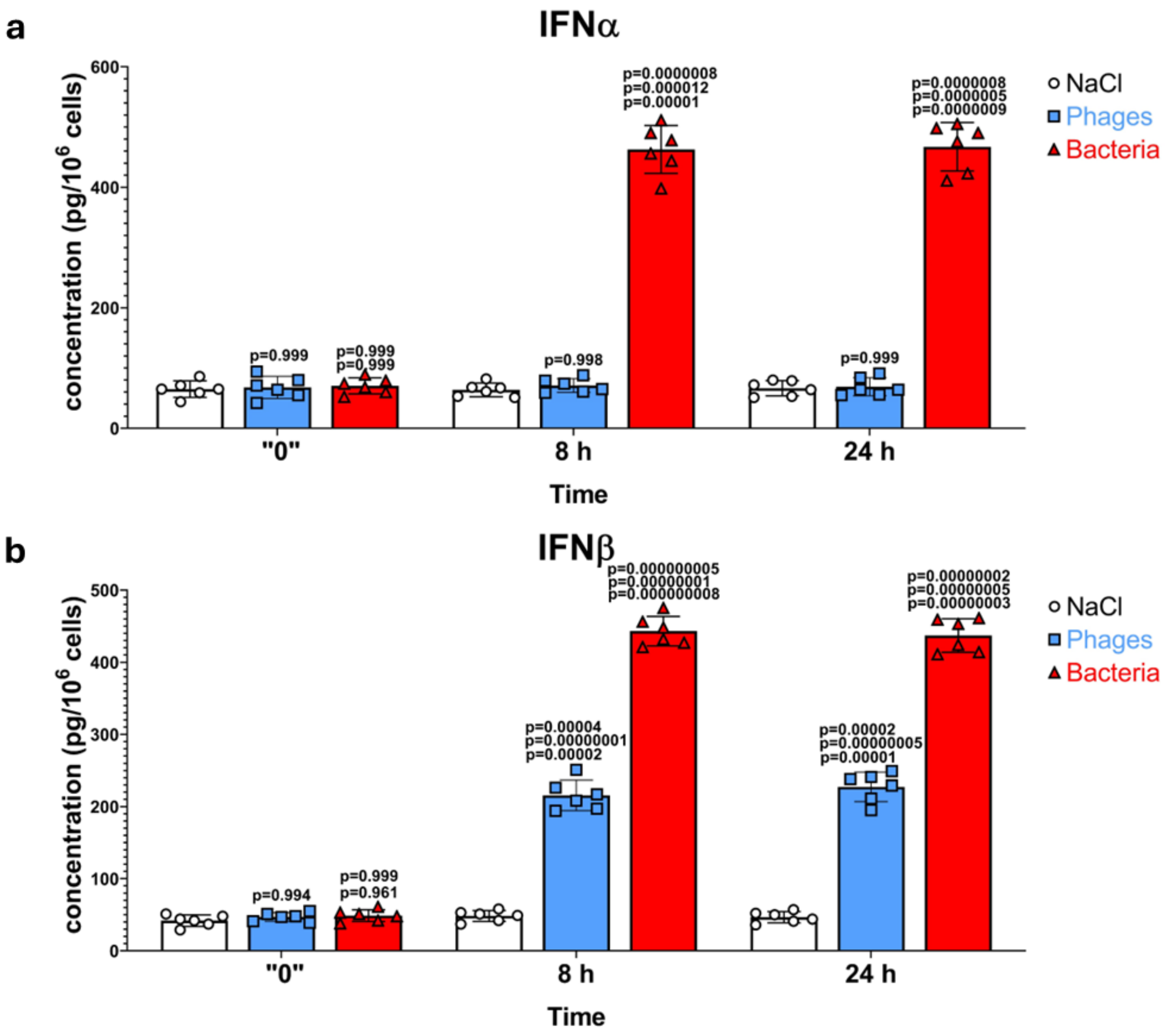

Supplementary Figure S4. Production of interferons in response of mouse splenocytes to phage and bacterial DNAs is similar to that in chickens infected with either phages or *S. enterica*. Levels of phosphorylated IFNα (a) and IFNβ (b) were determined by ELISA in lysates of mouse splenocytes 24 h after transfection with phage or bacterial DNA. Results correspond to samples obtained from cells (n=6; independent biological repeats) treated with NaCl (white columns with circles representing individual results), phage DNA (blue columns with squares), and bacterial DNA (red columns with triangles), and are mean values with error bars representing SD. For statistical analyses, the normality of the distribution of variables was checked with the Kolmogorov–Smirnov test and the homogeneity of the variances with Levene's test. Once both assumptions were met, the analysis was carried out using ANOVA and *post-hoc* Tukey's test. Otherwise (one or both assumption(s) was/were not met), the Kruskal–Wallis test and post-hoc Dunn test were applied (see the Source Data file for details). The *p* values obtained in the statistical analyses are indicated above the columns, indicating the comparison vs. the cells treated with NaCl (lower value), phage or bacterial DNA (middle values), and time 0 (upper values); consequently, particular columns are marked with one, two or three numbers. Statistically significant differences were considered when $p<0.05$. Detailed results demonstrated in this figure are included in the Source Data file.

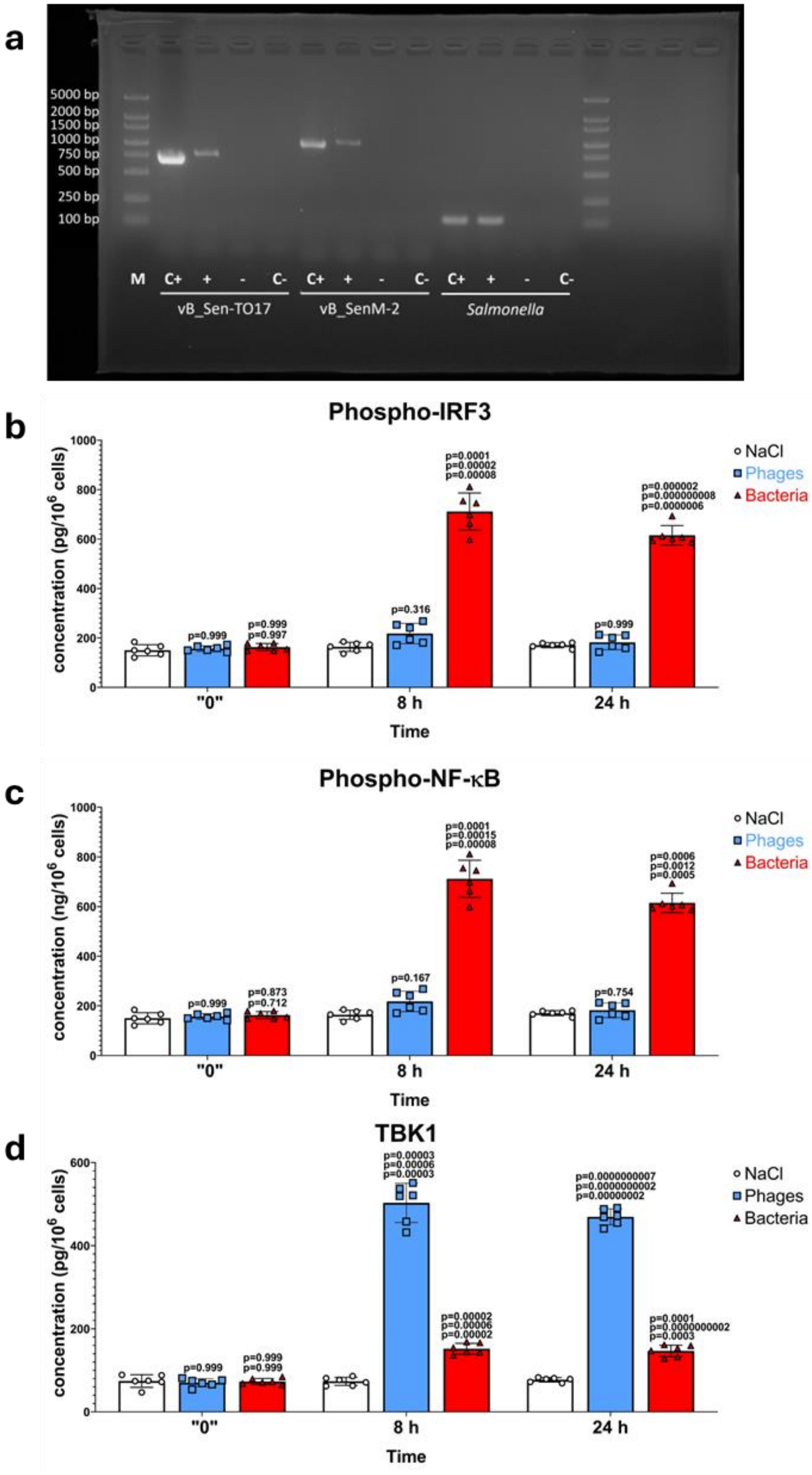
a
5000 bp
2000 bp
1500 bp
1000 bp
750 bp
500 bp
250 bp
100 bp
M C+ + - C- C+ + - C- C+ + - C-
vB_Sen-TO17
vB_SenM-2
Salmonella
b
Phospho-IRF3
concentration (pg/10^6 cells)
p=0.999
p=0.999
p=0.997
p=0.316
p=0.0001
p=0.00002
p=0.00008
p=0.999
p=0.000002
p=0.000000008
p=0.0000006
"0"
8 h
24 h
Time
NaCl
Phages
Bacteria
c
Phospho-NF-κB
concentration (ng/10^6 cells)
p=0.999
p=0.873
p=0.712
p=0.167
p=0.0001
p=0.00015
p=0.00008
p=0.754
p=0.0006
p=0.0012
p=0.0005
"0"
8 h
24 h
Time
NaCl
Phages
Bacteria
d
TBK1
concentration (pg/10^6 cells)
p=0.999
p=0.999
p=0.999
p=0.00003
p=0.00006
p=0.00003
p=0.00002
p=0.00006
p=0.00002
p=0.0000000007
p=0.0000000002
p=0.00000002
p=0.0001
p=0.0000000002
p=0.0003
"0"
8 h
24 h
Time
NaCl
Phages
Bacteria

Supplementary Figure S5. The immune response of mouse splenocytes to phage and bacterial DNAs is similar to that of chickens infected with either phages or *S. enterica*. Efficiency of transfection of mouse splenocytes by DNA from bacteriophages vB_Sen-TO17 and vB_SenM-2, and from *S. enterica* serovar Typhimurium was confirmed by PCR using DNA isolated from transfected cells, followed by agarose gel electrophoresis (a) (representative electropherograms are shown, with lane M containing molecular weight markers (with DNA sizes shown on the left), lanes C+ and C- indicating controls with and without purified phage/bacterial DNA, respectively, and lanes (+) and (-) indicating transfected and untransfected cells, respectively; each reaction was repeated 3 times giving the same results). Levels of phosphorylated IRF3 (b), phosphorylated NF-κB (c), and TBK1 (d) were determined by ELISA in lysates of mouse splenocytes 24 h after transfection with phage or bacterial DNA. In panels (b-d), results correspond to samples obtained from cells (n=6; independent biological repeats) treated with NaCl (white columns with circles representing individual results), phage DNA (blue columns with squares), and bacterial DNA (red columns with triangles), and are mean values with error bars representing SD. For statistical analyses (panels b-d), the normality of the distribution of variables was checked with the Kolmogorov–Smirnov test and the homogeneity of the variances with Levene's test. Once both assumptions were met, the analysis was carried out using ANOVA and *post-hoc* Tukey's test. Otherwise (one or both assumption(s) was/were not met), the Kruskal–Wallis test and post-hoc Dunn test were applied (see the Source Data file for details). The *p* values obtained in the statistical analyses are indicated above the columns, indicating the comparison vs. the cells treated with NaCl (lower value), phage or bacterial DNA (middle values), and time 0 (upper values); consequently, particular columns are marked with one, two or three numbers. Statistically significant differences were considered when $p<0.05$. Detailed results demonstrated in this figure are included in the Source Data file.

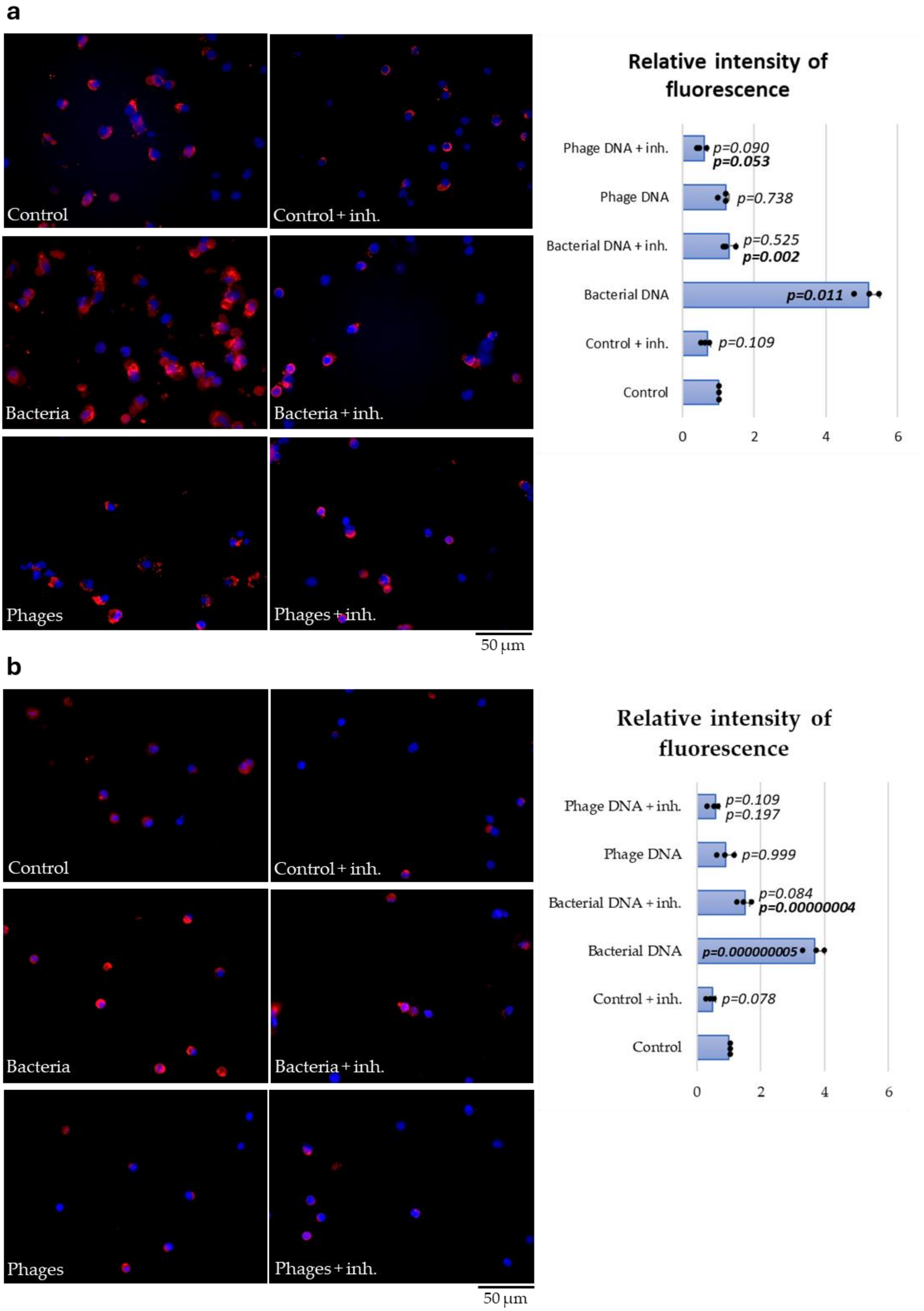
a
Control
Control + inh.
Bacteria
Bacteria + inh.
Phages
Phages + inh.
50 μm
Relative intensity of fluorescence
Phage DNA + inh.
p=0.090
p=0.053
Phage DNA
p=0.738
Bacterial DNA + inh.
p=0.525
p=0.002
Bacterial DNA
p=0.011
Control + inh.
p=0.109
Control
0
2
4
6
b
Control
Control + inh.
Bacteria
Bacteria + inh.
Phages
Phages + inh.
50 μm
Relative intensity of fluorescence
Phage DNA + inh.
p=0.109
p=0.197
Phage DNA
p=0.999
Bacterial DNA + inh.
p=0.084
p=0.00000004
Bacterial DNA
p=0.000000005
Control + inh.
p=0.078
Control
0
2
4
6

c

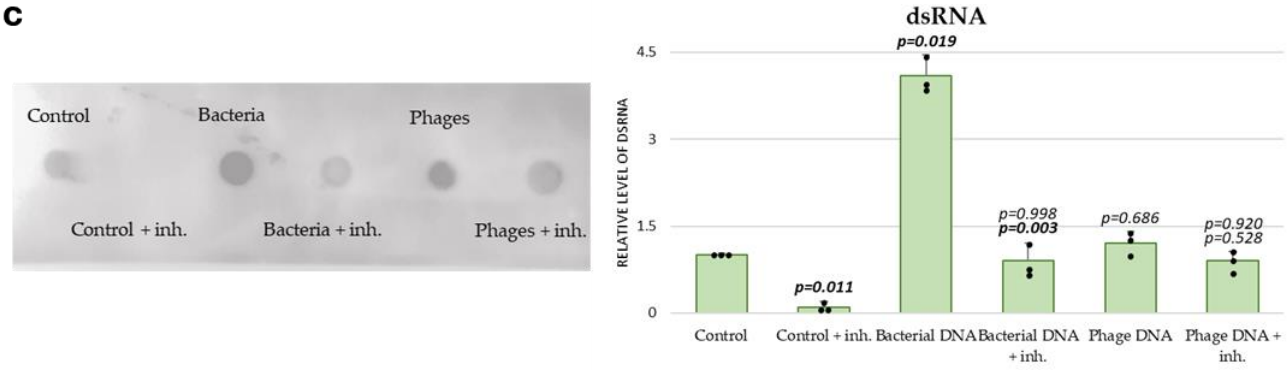

Supplementary Figure S6. The RNA polymerase III-synthesized dsRNA is abundant in mouse splenocytes after transfection with bacterial DNA, but not after transfection with phage DNA. dsRNA abundance in mouse splenocytes 24 h after transfection with bacterial or phage DNA was estimated by fluorescent microscopy, either without (a) or with (b) removing of erythrocytes, or by dot-blot (c), using specific anti-dsRNA antibody (a-c) and DAPI staining (a, b). The inhibitor of RNA polymerase III was added to final concentration of 25 μM (close to the IC_{50} value for the human enzyme, estimated as 27 μM) immediately before transfection. Examples of micrographs (a, b) and blots (c) are presented, and quantification of the results based on analyses of 100 randomly selected cells, repeated 3 times as independent biological experiments (a, b) or 3 independent dot-blot experiments (c) is presented near corresponding micrographs or blots as mean values (from 3 values of independent experiments, shown as dots) with error bars representing SD. For statistical analyses, the normality of the distribution of variables was checked with the Kolmogorov–Smirnov test and the homogeneity of the variances with Levene's test. Once both assumptions were met, the analysis was carried out using ANOVA and *post-hoc* Tukey's test. Otherwise (one or both assumption(s) was/were not met), the Kruskal–Wallis test and *post-hoc* Dunn test were applied (see the Source Data file for details). The p values obtained in the statistical analyses are indicated above the columns. When one number is shown, it corresponds to the statistical analysis vs. control. When two numbers are presented (in experiments with DNA-transfected cells treated with the RNA polymerase III inhibitor), the upper one corresponds to the statistical analysis vs. control, and the lower one to statistical analysis vs. analogues experiments without the inhibitor. Statistically significant differences were considered when $p<0.05$. Detailed results demonstrated in this figure are included in the Source Data file.

(a) Standard curve for chicken cyclic guanosine-adenosine phosphate synthetase (cGAS; cat. number: CK-bio-27451), range: 100 pg/ml – 4000 pg/ml, sensitivity: 10 pg/ml

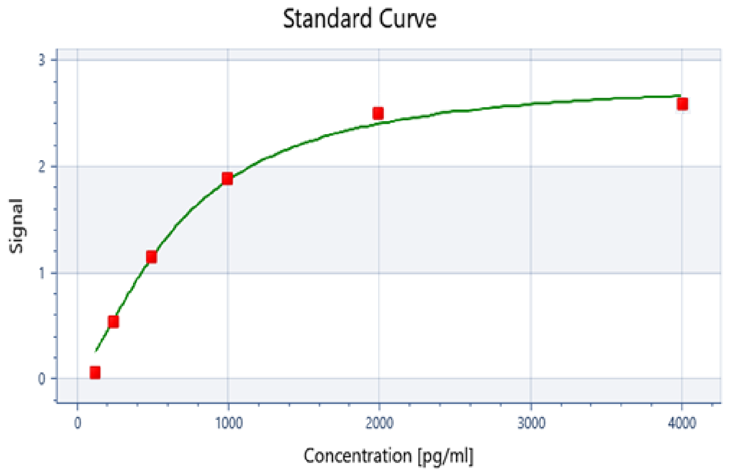

(b) Standard curve for chicken interleukin-1 receptor-associated kinase-1 (IRAK-1, cat. number: CK-bio-27453), range: 10 pg/ml – 600 pg/ml, sensitivity: 1 pg/ml

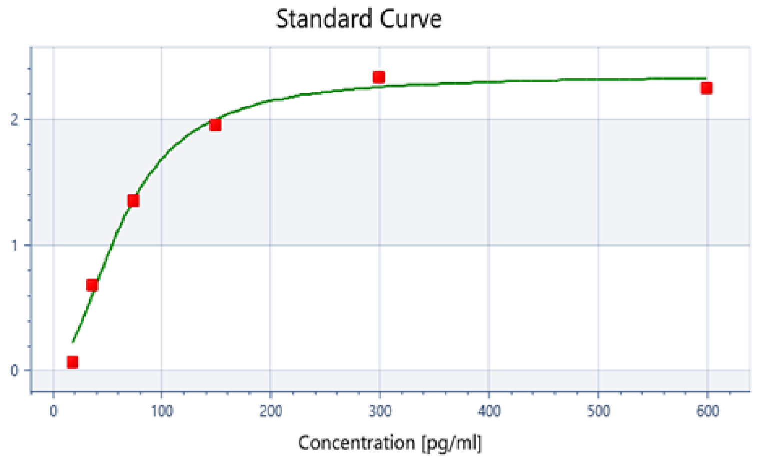

(c) Standard curve for chicken interleukin-1 receptor-associated kinase-4 (IRAK-4; cat. number: CK-bio-27452), range: 20 pg/ml – 2400 pg/ml, sensitivity: 10 pg/ml

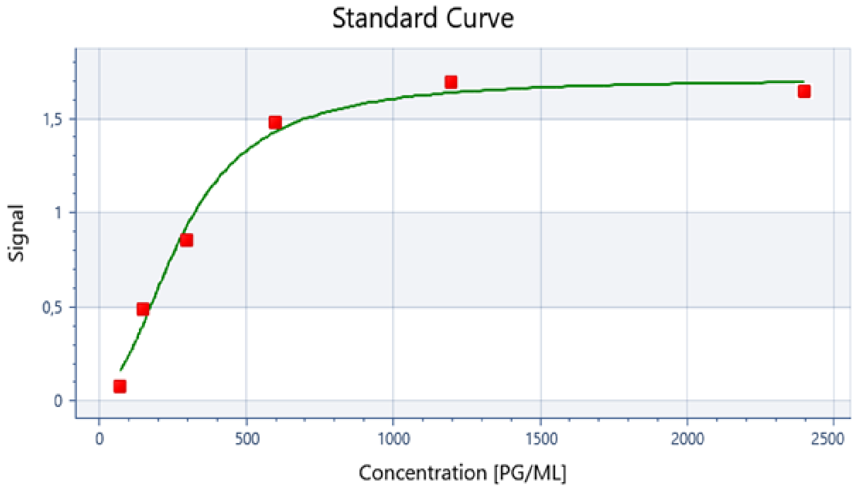

(d) Standard curve for chicken myeloid differentiation primary response protein MyD88 (MyD88; cat. number: CK-bio-27461), range: 100 pg/ml – 4000 pg/ml, sensitivity: 10 pg/ml

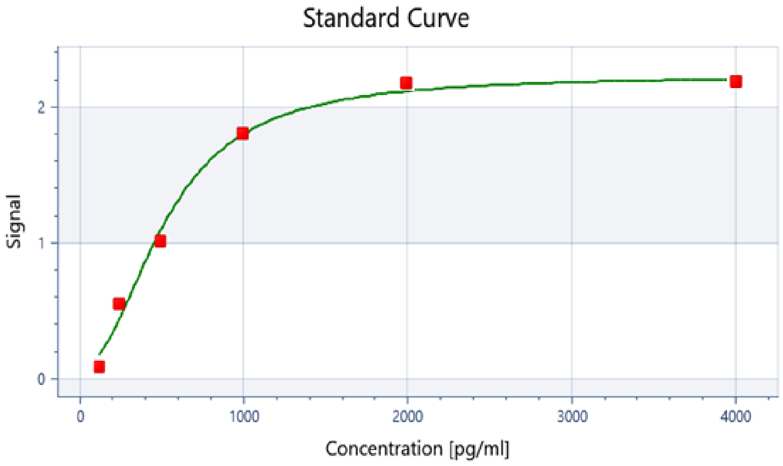

(e) Standard curve for chicken DEAD box protein 41 (DDX41; cat. number: CK-bio-22537), range: 20 pg/ml – 2000 pg/ml, sensitivity: 10 pg/ml

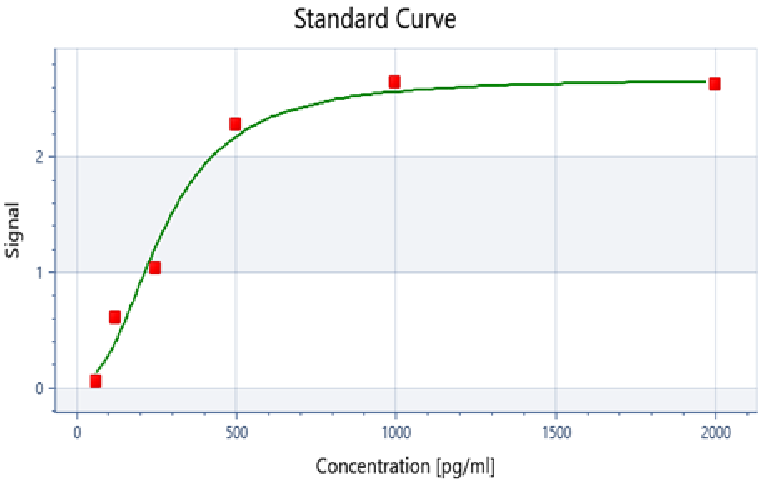

(f) Standard curve for chicken interferon-inducible protein 16 (IFI16; cat. number: CK-bio-22673), range: 1 ng/ml – 20 ng/ml, sensitivity: 0.1 ng/ml

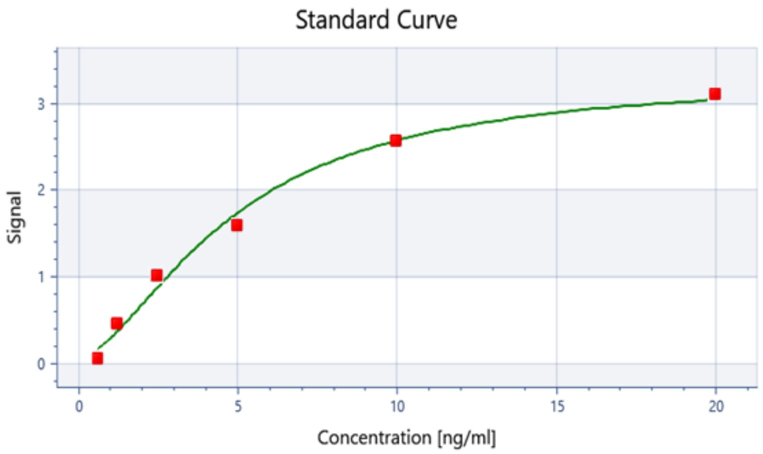

(g) Standard curve for chicken Tumor necrosis factor α (TNF-α; cat. number: CK-bio-18240), range: 20 pg/ml – 800 pg/ml, sensitivity: 10 pg/ml

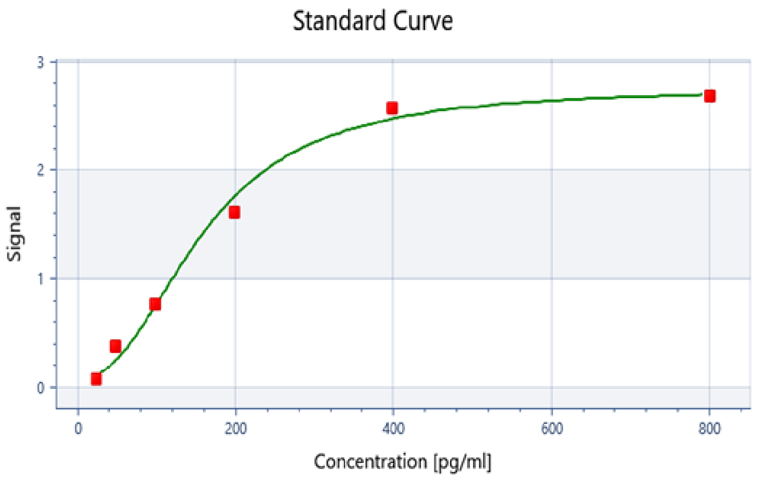

(h) Standard curve for chicken Interferon regulatory factor 7 (IRF7; cat. number: CK-bio-26639), range: 20 pg/ml – 1000 pg/ml, sensitivity: 10 pg/ml

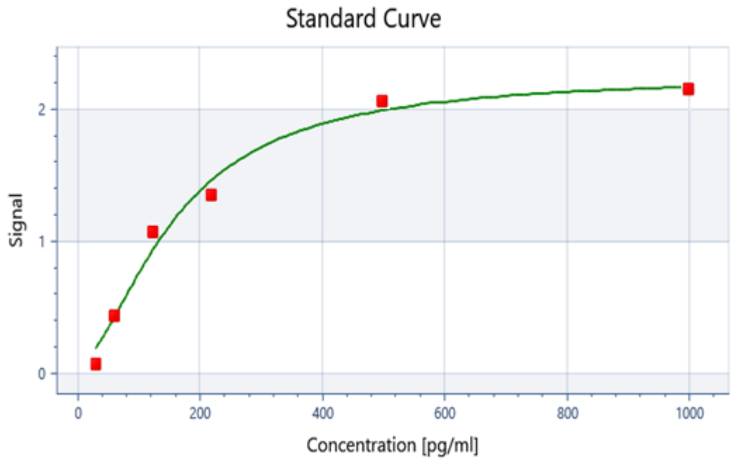

(i) Standard curve for chicken phospho- Interferon Regulatory Factor 3, phospho-IRF3 (Ser396; cat. number: CK-bio-29556), range: 1 ng/ml – 24 ng/ml, sensitivity: 0.1 ng/ml

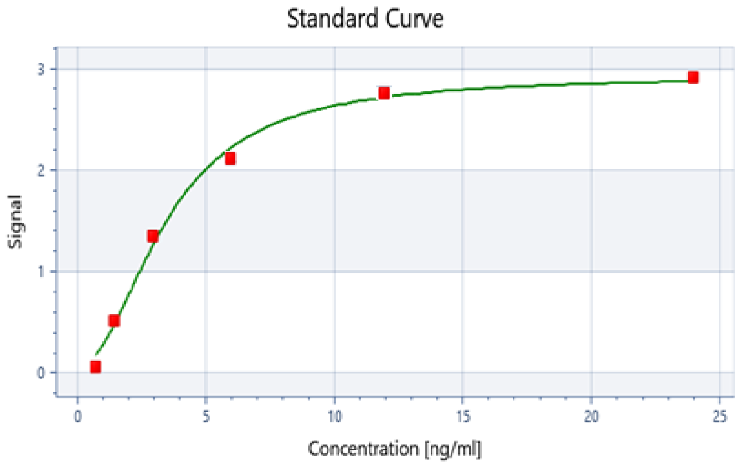

(j) Standard curve for Chicken phospho- Interferon Regulatory Factor 7 (phospho-IRF7 (Ser477; cat. number: CK-bio-22459), range: range: 1 ng/ml – 24 ng/ml, sensitivity: 0.1 ng/ml

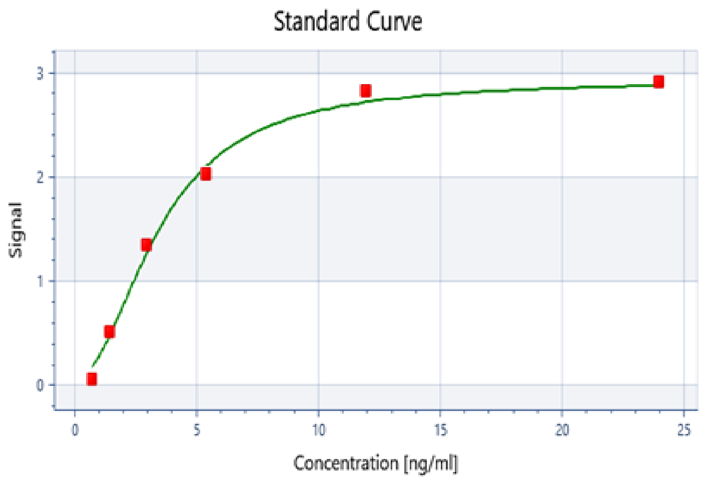

(k) Standard curve for chicken I-kappa-B kinase epsilon (IKKepsilon; cat. number: CK-bio-22816), range: 1 ng/ml – 32 ng/ml, sensitivity: 0.1 ng/ml

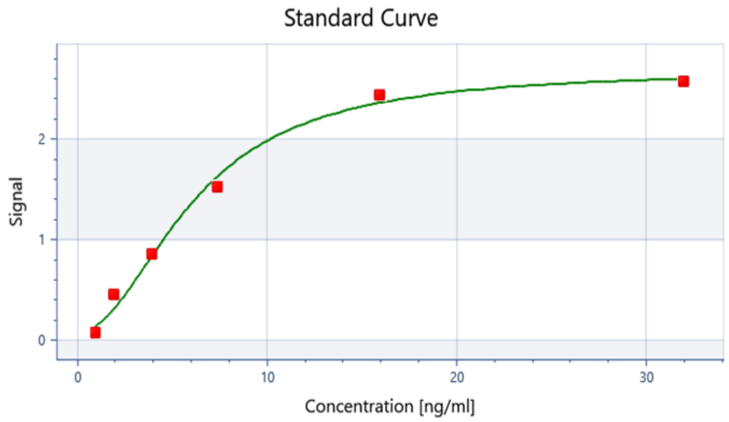

(l) Standard curve for chicken Nucleosome Assembly Protein 1 Like Protein 1(NAP1L1/NAP1; cat. number: CK-bio-26749), range: 20 pg/ml – 1000 pg/ml, sensitivity: 10 pg/ml

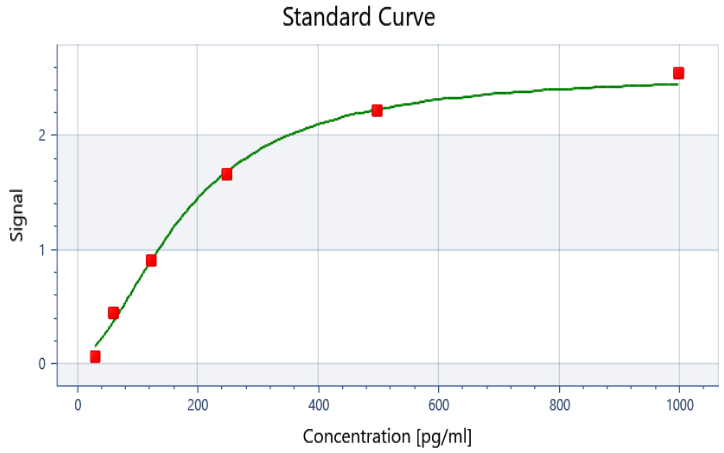

(m) Standard curve for chicken TANK-binding kinase 1-binding protein 1 (TBKBP1; cat. number: CK-bio-22537), range: 20 pg/ml – 2000 pg/ml, sensitivity: 10 pg/ml

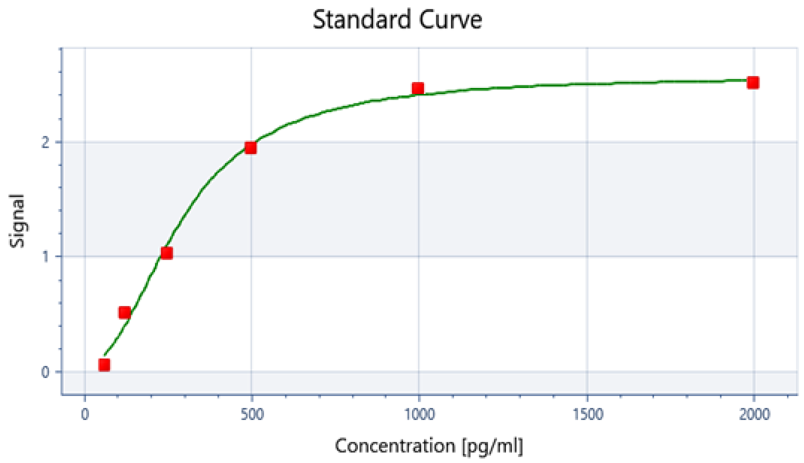

(n) Standard curve for chicken TRAF Family Member Associated NFKB Activator (TANK; cat. number: CK-bio-25271), range: 1 ng/ml – 20 ng/ml, sensitivity: 0.1 ng/ml

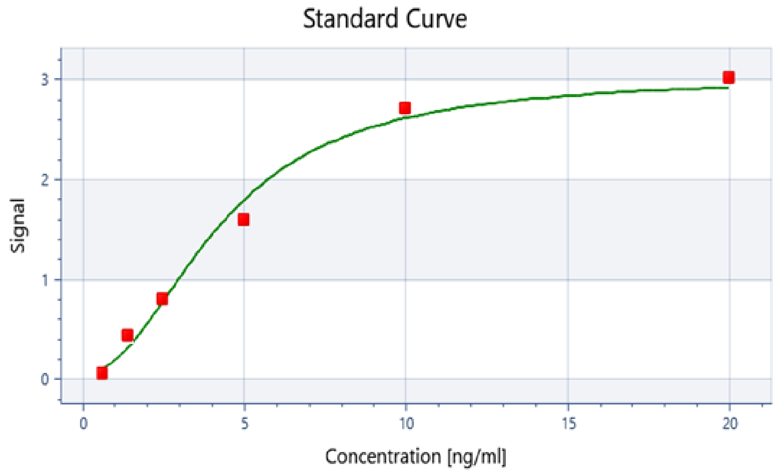

(o) Standard curve for chicken Cyclic Guanosine Monophosphate (cGMP; cat. number: CK-bio-24454), range: 1 nmol- 20 nmol/l, sensitivity: 0.1 nmol/l

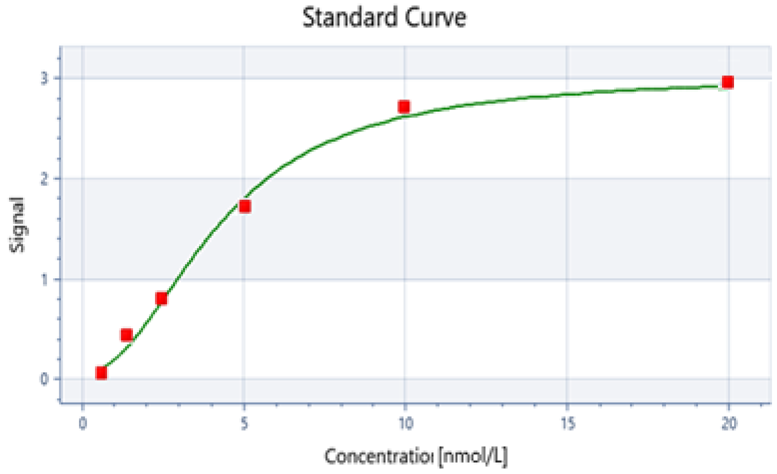

(p) Standard curve for chicken Interferon regulatory factor 3 (IRF3; cat. number: CK-bio-22057), range: 20 pg/ml – 1000 pg/ml, sensitivity: 10 pg/ml

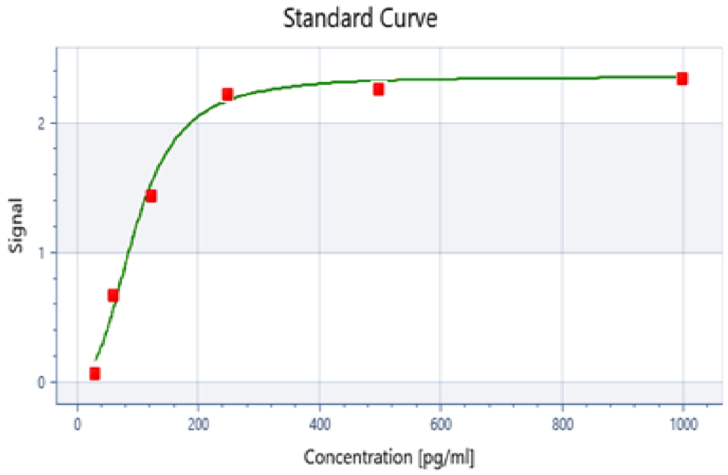

(q) Standard curve for chicken Interferon α (IFN-α; cat. number: CK-bio-18168), range: 10 pg/ml – 1000 pg/ml, sensitivity: 1 pg/ml

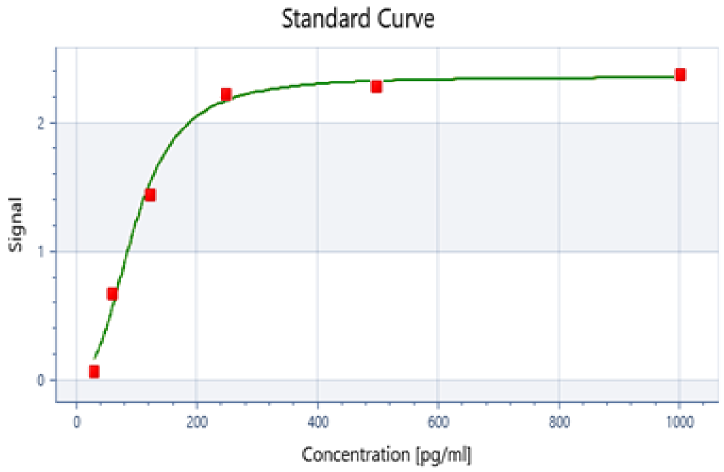

(r) Standard curve for chicken Nuclear factor-kappa B (NF-κB; cat. number: CK-bio-22695), range: 20 pg/ml – 1000 pg/ml, sensitivity: 10 pg/ml

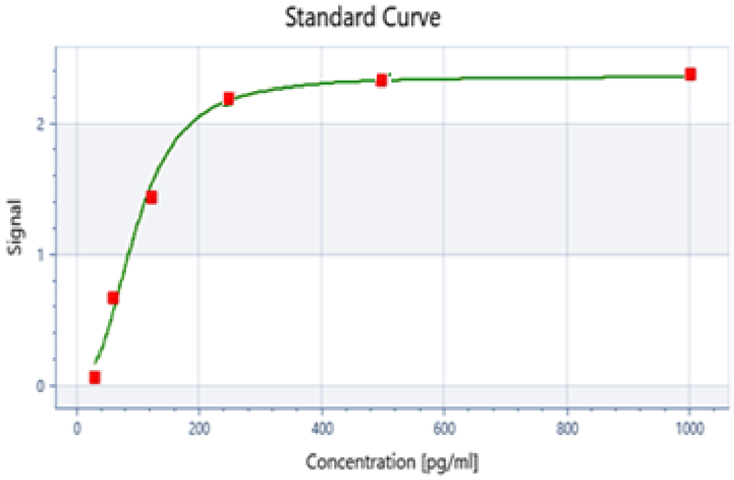

(s) Standard curve for chicken phospho- Nuclear Factor Kappa B, phosphoNFKB1 (S932; cat. number: CK-bio-29204), range: 1 ng/ml – 20 ng/ml, sensitivity: 0.1 ng/ml

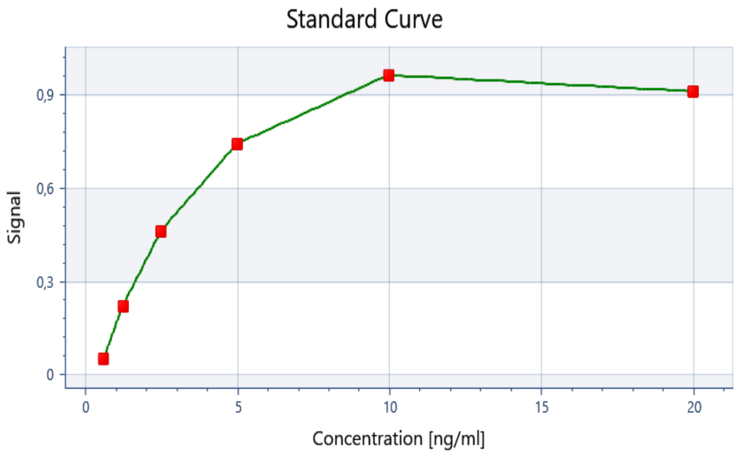

(t) Standard curve for chicken phospho- TANK-binding kinase 1, phosphoTBK1 (Ser172; cat. number: CK-bio-29739), range: 20 pg/ml – 1000 pg/ml, sensitivity: 10 pg/ml

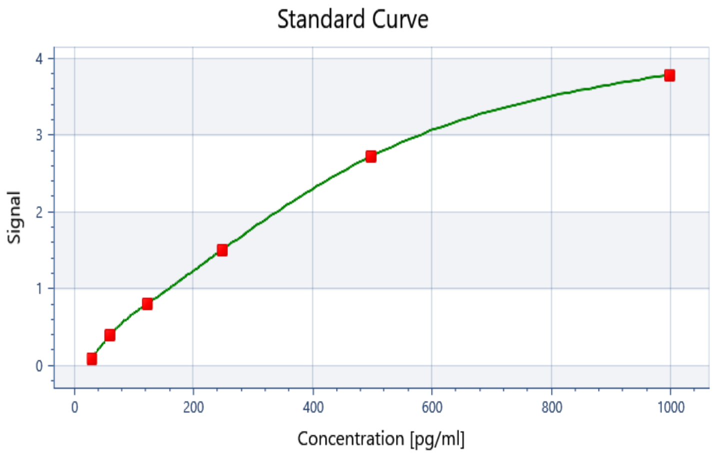

(u) Standard curve for chicken TANK-binding kinase 1 (TBK1; cat. number: CK-bio-28646), range: 0.5 ng/ml – 10 ng/ml, sensitivity: 0.1 ng/ml

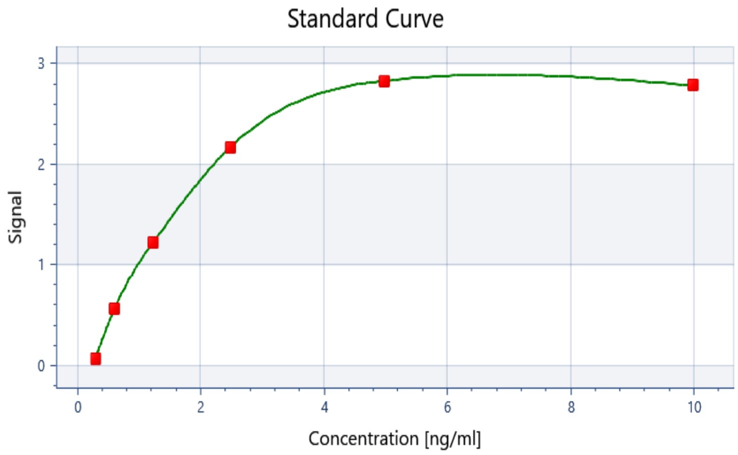

(v) Standard curve for chicken Toll-like receptor 9 (TLR9; cat. number: CK-bio-23737), range: 0.312 ng/ml – 20 ng/ml, sensitivity: 0.312 ng/ml

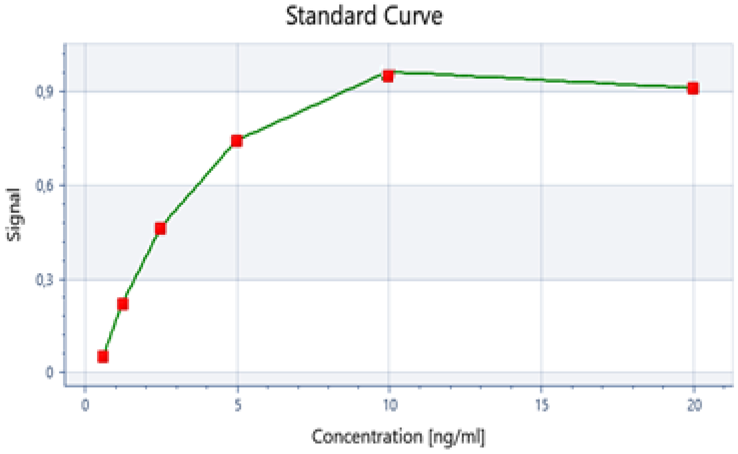

(w) Standard curve for chicken Transmembrane Protein 173 (TMEM173; cat. number: CK-bio-23792), range: 0.5 ng/ml – 12 ng/ml, sensitivity: 0.1 ng/ml

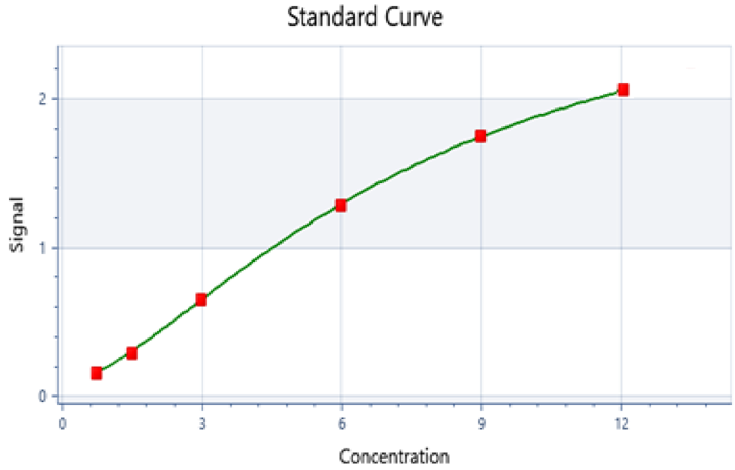

(x) Standard curve for chicken Toll Like Receptor 6 (TLR6; cat. number: CK-bio-23254), range: 10 pg/ml – 4000 pg/ml, sensitivity: 10 pg/ml

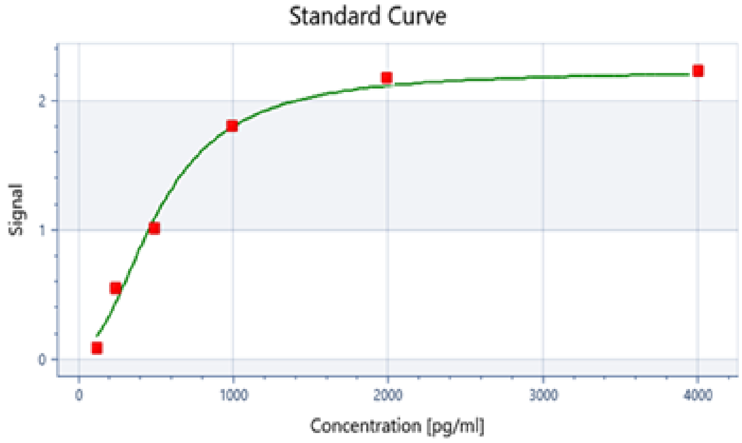

(y) Standard curve for chicken toll-like receptor 3 (TLR3; cat. number: CK-bio-23730), range: 20 pg/ml – 2400 pg/ml, sensitivity: 10 pg/ml

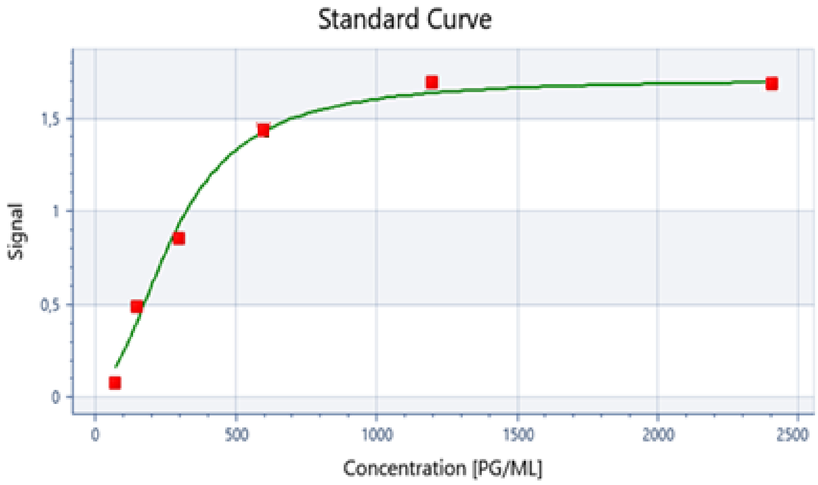

(z) Standard curve for chicken Interferon β (IFN-β/IFNB; cat. number: CK-bio-27460), range: 10 pg/ml – 800 pg/ml, sensitivity: 10 pg/ml

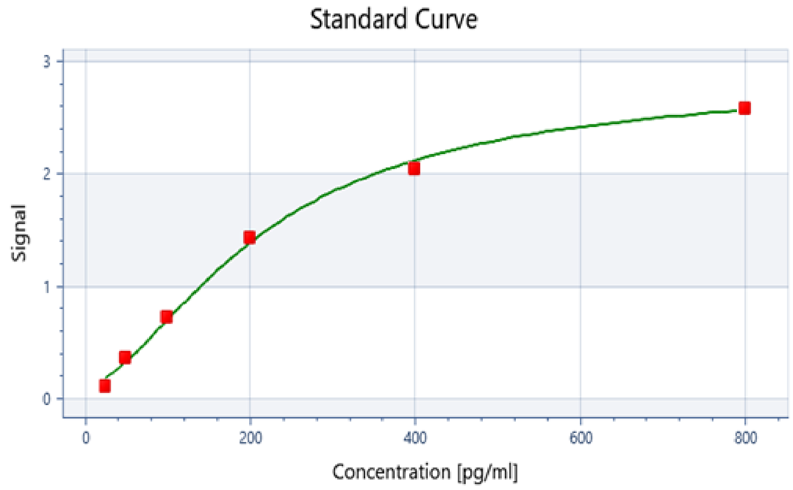

(aa) Standard curve for chicken Tumor Necrosis Factor receptor-associated factor 6 (TRAF 6; cat. number: Ck-bio-26795), range: 20 pg/ml – 2400 pg/ml, sensitivity: 10 pg/ml

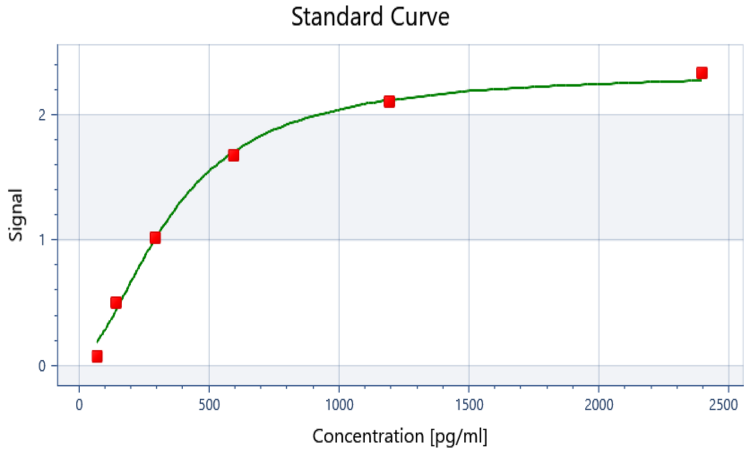

(bb) Standard curve for chicken Toll Like Receptor 4 (TLR4; cat. number: EIA06452Ch), range: 0.16-10 ng/mL, sensitivity: 0.071 ng/mL

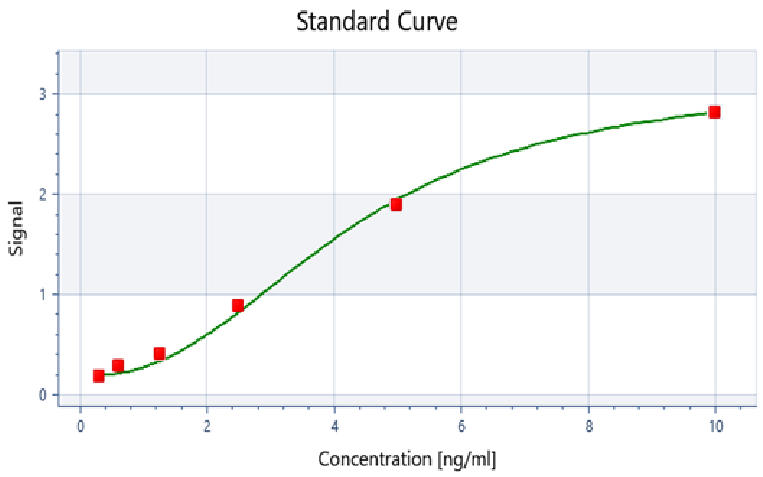

(cc) Standard curve for mouse phospho-IRF3 (Ser 379; cat. number: #54395), range: 100 pg/ml – 4000 pg/ml, sensitivity: 10 pg/ml

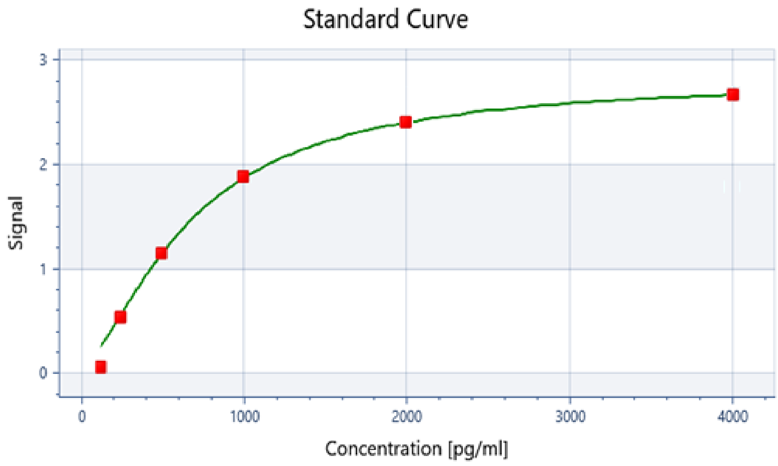

(dd) Standard curve for mouse NFKB-p65 (Nuclear Factor Kappa B p65 (cat. number: E-EL-M0838), range: 0.31 ng/ml – 20 ng/ml, sensitivity: 0.19 ng/ml

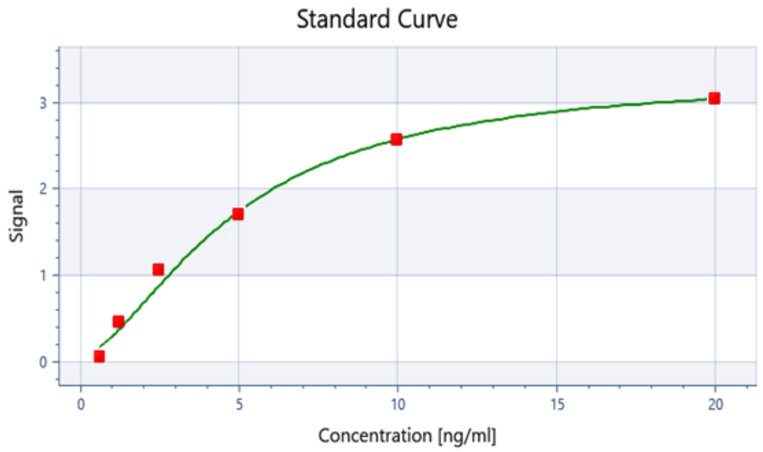

(ee) Standard curve for mouse TBK1 (TANK Binding Kinase 1; cat. number: MBS2533500), range: 20 pg/ml – 2000 pg/ml, sensitivity: 10 pg/ml

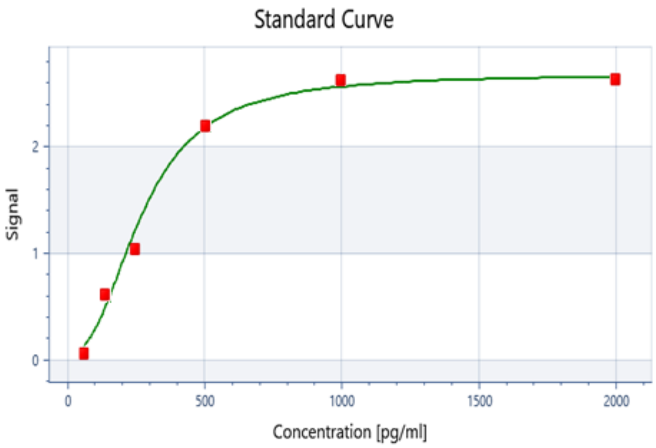

(ff) Standard curve for mouse Interferon Alpha (IFN-alpha; cat. number: MBS260421), range: 15.6 – 600 pg/ml, sensitivity: 5 pg/ml

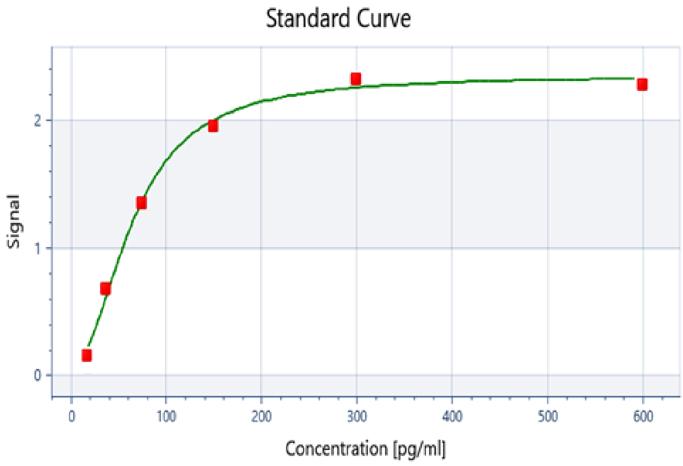

(gg) Standard curve for mouse Interferon Beta (IFN-beta; cat. number: MBS2502300), range: 15.63 – 1000 pg/ml, sensitivity: 9.38 pg/ml

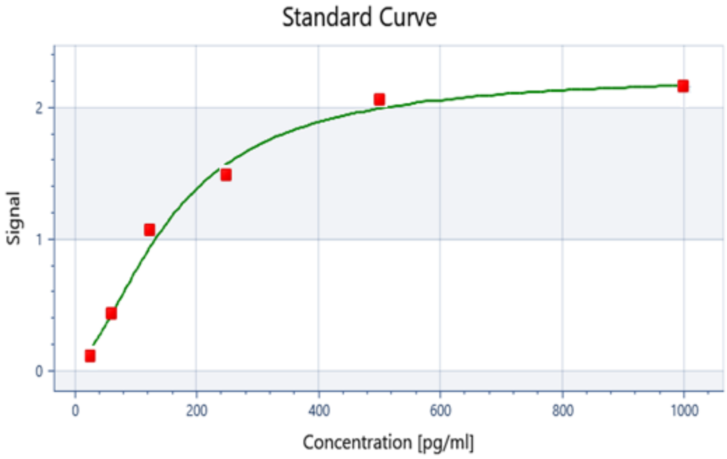

Supplementary Figure S7. Standard curves for validation of antibodies used in this work.
